# Prostaglandin E_2_ promotes *MYCN* non-amplified neuroblastoma cell survival *via* β-catenin stabilization

**DOI:** 10.1111/jcmm.12418

**Published:** 2014-09-30

**Authors:** Sepp R Jansen, Rian Holman, Ilja Hedemann, Ewoud Frankes, Carolina R S Elzinga, Wim Timens, Reinoud Gosens, Eveline S de Bont, Martina Schmidt

**Affiliations:** a Department of Molecular Pharmacology, University of GroningenGroningen, The Netherlands; b Department of Paediatrics, Department of Pediatric Oncology, University Medical Center Groningen, University of GroningenGroningen, The Netherlands; c Department of Pathology and Medical Biology, University Medical Center Groningen, University of GroningenGroningen, The Netherlands; d Groningen Research Institute for Asthma and COPD, GRIAC, University Medical Center Groningen, University of GroningenGroningen, The Netherlands

**Keywords:** neuroblastoma, prostaglandin E_2_, cyclic AMP, β-catenin

## Abstract

Amplification of *MYCN* is the most well-known prognostic marker of neuroblastoma risk classification, but still is only observed in 25% of cases. Recent evidence points to the cyclic adenosine monophosphate (cAMP) elevating ligand prostaglandin E_2_ (PGE_2_) and β-catenin as two novel players in neuroblastoma. Here, we aimed to define the potential role of PGE_2_ and cAMP and its potential interplay with β-catenin, both of which may converge on neuroblastoma cell behaviour. Gain and loss of β-catenin function, PGE_2_, the adenylyl cyclase activator forskolin and pharmacological inhibition of cyclooxygenase-2 (COX-2) were studied in two human neuroblastoma cell lines without *MYCN* amplification. Our findings show that PGE_2_ enhanced cell viability through the EP4 receptor and cAMP elevation, whereas COX-2 inhibitors attenuated cell viability. Interestingly, PGE_2_ and forskolin promoted glycogen synthase kinase 3β inhibition, β-catenin phosphorylation at the protein kinase A target residue ser675, β-catenin nuclear translocation and TCF-dependent gene transcription. Ectopic expression of a degradation-resistant β-catenin mutant enhances neuroblastoma cell viability and inhibition of β-catenin with XAV939 prevented PGE_2_-induced cell viability. Finally, we show increased β-catenin expression in human high-risk neuroblastoma tissue without *MYCN* amplification. Our data indicate that PGE_2_ enhances neuroblastoma cell viability, a process which may involve cAMP-mediated β-catenin stabilization, and suggest that this pathway is of relevance to high-risk neuroblastoma without *MYCN* amplification.

## Introduction

Neuroblastomas are heterogeneous tumours that vary to a large extent in prognosis and disease outcome and are responsible for 15% of all childhood cancer deaths [1]. Although recent studies have focused on the link between proliferation, differentiation and programmed cell death in neuroblastoma, understanding of the molecular mechanisms driving neuroblastoma heterogeneity is poor. The most extensively defined factor associated with neuroblastoma is the Myc oncoprotein family member *MYCN*, which is especially amplified in the most aggressive and often metastatic forms of neuroblastoma [2,3]. Although *MYCN* has important prognostic value, amplification is only observed in about 25% of neuroblastoma cases and it remains largely to be defined what other factors contribute to high-risk neuroblastoma.

Expression of cyclooxygenase-2 (COX-2) and prostaglandin E_2_ (PGE_2_) have been found increased in a variety of malignant tumours, including neuroblastoma [4,5] and pharmacological inhibition of COX-2 has been shown to attenuate cell cycle progression in malignant cells [6–9]. PGE_2_ is produced by a multistep enzymatic process in which the rate-limiting step is mediated by COX enzymes. PGE_2_ binds to its membrane bound E-type prostanoid receptors, of which prostanoid receptors type 2 and 4 are known to couple to Gα_s_ and are thereby able to increase intracellular cyclic adenosine monophosphate (cAMP) levels. cAMP is involved in the regulation of diverse cellular processes, including regulation of cytoskeletal dynamics, cellular differentiation, proliferation and programmed cell death in a variety of cells including neural-like cells [10,11].

Of particular interest are recent research lines that focus on molecular interactions between PGE_2_, cAMP and β-catenin. β-catenin contributes to other malignancies such as hepatocellular carcinoma and colorectal carcinoma and its role in paediatric malignancies is well documented [12]. Also, its role in normal physiological development of pluripotent cells from the neural crest has been well-established [13–15]. Regarding neuroblastoma, β-catenin expression is increased in *MYCN* non-amplified neuroblastoma cell lines and β-catenin target gene transcription is increased in neuroblastoma tumours without *MYCN* amplification [16].

Distinct pools of β-catenin exhibit distinct cellular functions. β-Catenin associates with membrane junctional complexes where it binds to cadherins and α-actin. Free cytosolic β-catenin is rapidly tagged for proteasomal degradation by a multiprotein destruction complex comprised of the kinases glycogen synthase kinase 3β (GSK3β), casein kinase 1 and adaptor proteins like axin2, which is the limiting component in the assembly of this complex [17–19]. Stabilized β-catenin translocates to the nucleus, where it activates transcription of TCF/Lef target genes. The result is expression of mitogenic and survival genes including Myc oncogene family members [20] and cyclin D1 [21]. Interestingly, PGE_2_ has been shown to enhance β-catenin nuclear localization *via* dissociation of GSK3β from axin by Gα_s_ [22] and by activating protein kinase A (PKA) [23]. Activated PKA can directly phosphorylate β-catenin at residue ser675 [24] and GSK3β at residue ser9 [10,25,26].

In this paper, we aim to identify the contribution of a molecular link between PGE_2_ and β-catenin to cell proliferation and inhibition of apoptosis, independent of *MYCN* amplification.

## Materials and methods

### Cell culture

Human neuroblastoma cell lines SK-N-AS and SK-N-SH were obtained from ATCC (Manassas, VA, USA). Both cell lines are of epithelial morphology. Cells were maintained in DMEM (1.0 g/l glucose, HEPES) supplemented with 10% v/v heat-inactivated FCS, non-essential amino acids and antibiotics (penicillin 100 U/ml, streptomycin 100 μ/ml) in a humidified atmosphere of 5% CO_2_ at 37°C. Cells were washed with HBSS (400 mg/l KCl, 60 mg/l KH_2_PO_4_, 8 g/l NaCl, 350 mg/l NaHCO_3_, 50 mg/l Na_2_HPO_4_·H_2_O, 1 g/l glucose, pH 7.4), dissociated from the plate with trypsin EDTA and seeded in appropriate cell culture plate format. Cells were serum-deprived for 24 hrs before stimulation. Inhibitors (XAV939, celecoxib and niflumic acid) or antagonists (AH6809 and L-161,982) were added 30 min. prior to stimulation with PGE_2_.

### Cell viability assay

Experiments were performed in 24-well cell culture plates. Prior to measurement, cells were washed with calcium containing HBSS (400 mg/l KCl, 60 mg/l KH_2_PO_4_, 8 g/l NaCl, 140 mg/l CaCl_2_, 100 mg/l MgCl_2_·6H_2_O, 100 mg/l MgSO_4_·7H_2_O, 90 mg/l Na_2_HPO_4_·7H_2_O, 1 g/l glucose, pH 7.4) and then incubated with 5% v/v AlamarBlue (Invitrogen, Carlsbad, CA, USA) followed by fluorescence spectrophotometry. Treated cultures were normalized to control cultures.

### cAMP assay

Experiments were performed in 24-well cell culture plates. When indicated, cells were pre-incubated with niflumic acid for 2 hrs. Cells were incubated in serum-free DMEM supplemented with 0.1 mM 3-Isobutyl-1-methylxanthine for 10 min at 37°C with indicated stimuli. A radioactive competitive binding assay was used to determine cAMP levels, as described earlier [27–30].

### Colony formation assay

Cells were seeded in six-well plates. Cells were incubated in DMEM for 14 days with indicated stimuli. Medium was refreshed every 3 days. Cells were fixed with paraformaldehyde (PFA) and stained with 0.05% Crystal Violet. Plates were photographed and confluency was quantified using ImageJ software.

### PGE_2_ ELISA

Cells were cultured in 24-well plates and PGE_2_ production was measured in culture medium from overnight cultures incubated with niflumic acid using the PGE_2_ ELISA assay from Cisbio (Codolet, France).

### Fluorescence-activated cell sorting

Cells were cultured on 60 mm plates and incubated for 24 hrs with designated stimuli. For cell cycle analysis, cells were incubated for 30 min. with 30 μM bromodeoxyuridine (BrdU). Cells were dissociated from the plate with accutase, fixed with 95% EtOH and DNA was denatured using 2M HCl. Cells were incubated with anti-BrdU FITC-conjugated antibody, propidium iodide (PI) and RNAse A. A total of 40,000 stained cells in FACS buffer (PBS containing 1% v/v BSA, 20 mM EDTA) were analysed in FACSCalibur (BD Biosciences, Franklin Lakes, NJ, USA). ModFit LT software was used to determine the distribution of cells over the cell cycle phases designated G0/G1, S and G2/M. For detection of apoptosis, cells were washed with binding buffer (140 mM NaCl, 4 mM KCl, 0.75 mM MgCl_2_, 10 mM HEPES). A total of 400,000 cells were resuspended in ice-cold binding buffer supplemented for 15 min. with 2 mM CaCl_2_, PI (1 μg/ml) and FITC-conjugated annexin V antibody (IQ Products, Groningen, The Netherlands). A total of 10,000 stained cells were analysed by Kaluza Flow Analysis Software (Beckman Coulter, Brea, CA, USA).

### JC-1 mitochondrial membrane polarization assay

Cells were seeded in 24-well plates and incubated with designated stimuli for 24 hrs and mitochondrial membrane polarization was measured using the Mitochondrial Staining Kit (Sigma-Aldrich, Munich, Germany).

### Isolation of mRNA and real-time PCR analysis

Total mRNA extraction was performed with the NucleoSpin RNA II Kit (Machery-Nagel, Düren, Germany). cDNA was acquired using reverse transcription by AMV Reverse Transcriptase Kit (Promega, Madison, WI, USA). qPCR was performed with the Illumina Eco Personal qPCR System (Westburg, Leusden, The Netherlands). Cycle parameters (30 sec. each): denaturation at 94°C, annealing at 60°C and extension at 72°C. Target genes were normalized to the geometric mean of reference genes GAPDH, SDHA and YWHAZ [29]. Primer sequences are listed in Table 1.

**Table 1:** Primer sequences

Gene	Forward	Reverse
MYCN	CTAAACGTTGGTGACGGTTG	GGTATCAAATGGCAAACCCC
CCND1	ATGCCAACCTCCTCAACGAC	GGCTCTTTTTCACGGGCTCC
EP1	TTGGGTGTACATCCTACTGC	TGTGCTTAGAAGTGGCTGAG
EP2	TGGCTATCATGACCATCACC	TCCTTTCGGGAAGAGGTTTC
EP3	TAGCTCTTCGCATAACTGGG	GTTGCAGGAAAAGGTGACTG
EP4	GAACATCCTGGCTTTTGAGC	TGTGACCACAATCCTCTGTC
GAPDH	CCAGCAAGAGCACAAGAGGA	GAGATTCAGTGTGGTGGGGG
SDHA	TGGGAACAAGAGGGCATCTG	CCACCACTGCATCAAATTCATG
WYHAZ	ACTTTTGGTACATTGTGGCTTCAA	CCGCCAGGACAAACCAGTAT

### Transfection

Cells grown to 60% confluence were transfected in serum- and antibiotics-free DMEM with plasmid DNA [TOPflash, FOPflash, renilla luciferase (Upstate Biotechnology, Charlottesville, VA, USA), β-catenin^S33Y^ (AddGene plasmid 19286, Cambridge, MA, USA) [30], pcDNA3 vector] using X-tremeGENE 9 DNA transfection reagent (Roche Applied Science, Penzberg, Germany).

### TOP flash assay

TOPflash- or FOPflash-transfected cells were subjected to stimulation in serum-free DMEM for 16 hrs, and luciferase activity was assayed *via* the Dual Reporter luciferase assay system (Promega).

### Preparation of nuclear fractions

Cell were seeded in 100 mm cell culture dishes and dissociated with a cell scraper. Cells were incubated with hypotonic buffer (25 mM HEPES pH 7.5, 10 mM KCl, 1 mM EDTA, 0.2 mM Na_3_VO_4_, 50 mM NaF, β-glycerophosphate, 10% v/v glycerol, pepstatin A, leupeptin, apoprotinin) for 10 min. on ice and 15 min. with 0.1% v/v IGEPAL added. After centrifugation at 3000 × g for 3 min., the supernatant (cytosolic fraction) was collected and pellets were resuspended in ice-cold cell extraction buffer (25 mM HEPES pH 7.5, 420 mM NaCl, 10% w/v sucrose, 10 mM KCl, 1 mM EDTA, 10% v/v glycerol, 50 mM NaF, 0.2 mM Na_3_VO_4_, β-glycerophosphate, pepstatin A, leupeptin, apoprotinin) for 30 min. After centrifugation at 14,000 × g for 10 min., supernatant (nuclear fraction) was collected.

### Immunoblotting

Cells were lysed in ice-cold RIPA buffer supplemented with phosphatase and protease inhibitors (aprotinin, leupeptin, pepstatin A, Na_3_VO_4_, NaF, β-glycerophosphate). Equal amounts of protein were subjected to electrophoresis on polyacrylamide gels and transferred to nitrocellulose membranes. Membranes were blocked with 5% w/v BSA or 5% w/v milk in tris-buffered saline with 0.1% v/v Tween-20. Protein expression was determined by specific primary and horseradish peroxidase (HRP)-conjugated secondary IgGs. Antibodies used are listed in Table 2. Protein expression was visualized by ECL in the Syngene G:BOX HR iChemi gel documentation system (Syngene, Cambridge, UK). Band was quantified by densitometry using ImageJ software.

**Table 2:** Primary antibodies

Antibody	Source (catalogue number)	Dilution
Cyclin D1	Cell Signaling (2926)	1:1000
Histone H3	Cell Signaling (9715)	1:500
PARP	Cell Signaling (9542)	1:1000
p-GSK3α/β (ser21/9)	Cell Signaling (9331)	1:500
p-β-Catenin (ser675)	Cell Signaling (4176)	1:2000
α-Tubulin	Millipore (05-829)	1:2000
Active β-Catenin	Millipore (05-665)	1:1000
β-Catenin (total)	BD Biosciences (610153)	1:2000

### Immunofluorescence

Cells were grown on LabTek II Chamber Slides (Thermo Fisher Scientific, Marietta, OH, USA), fixed with PFA and permeabilized with 0.3% w/v Triton X-100 in cytoskeletal buffer (10 mM Tris, 150 mM NaCl, 5 mM EGTA, 5 mM MgCl_2_, 5 mM glucose, pH 6.1). Cells were blocked using 1% w/v BSA and 2% v/v donkey serum in CytoTBS-T (20 mM Tris, 154 mM NaCl, 2 mM EGTA, 2 mM MgCl_2_, 0.1% v/v Tween-20, pH 7.2). p-β-Catenin (ser675) IgG was applied overnight, secondary FITC-conjugated donkey anti-rabbit IgG (Jackson Laboratories, Bar Harbor, ME, USA) was applied for 3 hrs. Nucleus was visualized with 1 μg/ml Hoechst 33342 (Invitrogen). Slides were mounted with ProLong® Gold Antifade Reagent (Life Technologies, Marietta, OH, USA). Images were captured with a Leica DM4000 B Fluorescence microscope (Leica Microsystems, Wetzlar, Germany) equipped with a Leica DFC 345 FX camera.

### Study population and samples

Formalin-fixed paraffin-embedded tumour samples from 31 neuroblastomas were analysed. All samples were collected at our own hospital between 1984 and 2012 according to the Research Code of the University Medical Center Groningen (https://www.umcg.nl/en/research/researchers/general/researchcode/pages/default.aspx) and national ethical and professional guidelines (Code of Conduct; Dutch Federation of Biomedical Scientific Societies; http://www.federa.org). Tumour specimens were subdivided based on risk group assessment in low risk, medium risk and high risk according to Children's Oncology Group risk groups (Table 3).

**Table 3:** The Children's Oncology Group (COG) risk group classification

Risk group	Stage (INSS)	Age	Histology	DNA Ploidy	MYCN
Low risk	Stage 1	Any	Any	Any	Any
	Stage 2A/B	<12 months	Any	Any	Any
	Stage 2A/B	>12 months	Any	Any	Non-Amplified
	Stage 4S	<12 months	Favourable	Hyperdiploid	Non-Amplified
Medium risk	Stage 3	<12 months	Any	Any	Non-Amplified
	Stage 3	>12 months	Favourable	Any	Non-Amplified
	Stage 4	<12 months	Any	Any	Non-Amplified
	Stage 4S	<12 months	And/or Unfavourable	And/or Normal	Non-Amplified
High risk	Stage 2A/B	>12 months	Any	Any	Amplified
	Stage 3	Any	Any	Any	Amplified
	Stage 3	>18 months	Unfavourable	Any	Any
	Stage 4	Any	Any	Any	Amplified
	Stage 4	>18 months	Any	Any	Any
	Stage 4	>12 months <18 months	And/or Unfavourable	And/or Normal	And/or Amplified
	Stage 4S	<12 months	Any	Any	Amplified

### Fluorescence *in situ* hybridization

Fluorescence *in situ* hybridization was performed to exclude *MYCN*-amplified tumours from the study population. Tissue sections were hybridized with Vysis LSI N-MYC (2p24) SpectrumGreen/CEP2 SpectrumOrange Probe (Abbott Molecular, Green Oaks, IL, USA). The LSI N-MYC (2p24) probe hybridizes to the 2p24 region and contains sequences that flank both 5′ and 3′ ends of the *MYCN* gene. The CEP2 (2p11.1-q11.1) probe hybridizes to alpha satellite sequences specific to chromosome 2. Images were captured using a Leica DMRA2 Fluorescence microscope (Leica Microsystems) equipped with a Leica DC350F camera. The number of spots in 20 adjacent tumour cells was counted for *MYCN* and *CEP2*. When ratio between the two signals exceeded 2, the tumour was scored as *MYCN* amplified.

### Immunohistochemistry

For determination of β-catenin protein expression, deparaffinized tumour sections were incubated with anti-β-catenin IgG (1:25 in 1% v/v goat serum, 1% v/v human serum) overnight and subsequent with HRP-conjugated rabbit antimouse IgG (1:1000; DAKO, Glostrup, Denmark). Staining was performed with 0.5 mg/ml 3,3′-diaminobenzidine in 30 mM imidazole containing 0.03% v/v H_2_O_2_ and 1 mM EDTA, pH 7.0. As a negative control, the primary antibodies were omitted. For each sample, expression was quantified by measuring the average staining intensity on a scale of 256 channels using ImageJ software in four different random selected regions within the tumour (using haematoxylin and eosin-stained sections). Differences between the groups were tested for significance using the Kruskal–Wallis test. All procedures were performed blind.

### Reagents

BrdU, mouse anti-BrdU antibody, PI, L-161,982 and celecoxib were purchased from Sigma-Aldrich. 16,16-dimethyl-PGE_2_, XAV939, AH6809 and forskolin were from Tocris Bioscience (Bristol, UK). Niflumic acid was from Cayman Chemical (Ann Arbor, MI, USA). All other chemicals were of analytical grade.

### Statistics

Data represent means ± SEM, from *n* separate experiments. Normality and equal variance were evaluated by Shapiro–Wilk test and *f*-test. Statistical significance of differences was evaluated by Student's *t*-test and one-way or two-way anova followed by a Tukey multiple comparison test. Non-parametric data were evaluated with the Kruskal–Wallis test followed by Dunn's multiple comparison test. Differences were considered to be statistically significant when *P* < 0.05.

## Results

### cAMP and PGE_2_ enhance neuroblastoma cell viability

**Fig. 1:**
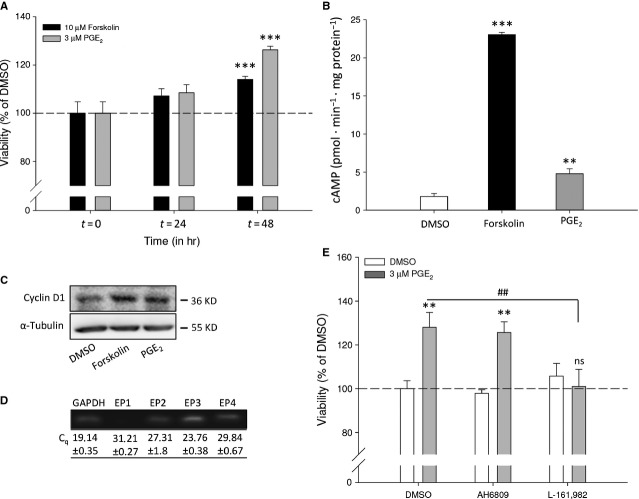
Effect of PGE_2_ and forskolin on SK-N-AS cell viability. (**A**) Cell viability after 24- and 48-hr incubation with 10 μM forskolin or 3 μM PGE_2_. (**B**) cAMP formation under basal (1.8 ± 0.38 pmol/min/mg/protein), forskolin (23.04 ± 0.31/pmol/min/mg protein) and PGE_2_ (4.79 ± 0.64 pmol/min/mg protein)-treated conditions. (**C**) Representative Western blot images showing expression of cyclin D1 after 8-hr incubation with forskolin or PGE_2_. (**D**) qRT-PCR detection of EP1-4 mRNA expression (C_q_ values) and representative bands on agarose gel. (**E**) Cell viability after 48-hr incubation with 10 μM AH6809 and 3 μM L-161,982 in combination with PGE_2_. Data represent mean ± SE of the mean of four separate experiments. *P < 0.05, **P < 0.01, ***P < 0.001 compared to DMSO-treated cells.

To study the effects of cAMP elevation on *MYCN* non-amplified neuroblastoma cell viability, SK-N-AS cells were incubated for the indicated periods of time with the direct adenylyl cyclase activator forskolin or PGE_2_ (Fig. 1A). Forskolin or PGE_2_ enhanced time-dependently cellular viability compared to control treated cells. To determine that PGE_2_ could act *via* elevation of cAMP, we measured intracellular cAMP levels. Cells show increase in cAMP levels after incubation with forskolin compared to basal. Similar, 16,16-dimethyl-PGE_2_ (in this paper referred to as PGE_2_) induces cAMP elevation (Fig. 1B).

In addition, expression of cyclin D1, a cell cycle regulatory protein involved in G1/S-phase transition, is increased by both forskolin and PGE_2_ (Fig. 1C).

Prostaglandin E_2_ mediates its effects through activation of four subtypes of E-type prostanoid receptors, termed EP1-4. Of these receptors, EP2 and EP4 are known to be Gα_s_-coupled and enhance intracellular cAMP. SK-N-AS neuroblastoma cells express all four receptor subtypes (Fig. 1D). To study which receptor is involved in enhancement of cell viability by PGE_2_, the EP2 and EP4 receptor were antagonized using AH6809 and L-161,982 respectively. AH6809 did not affect PGE_2_-enhanced cell viability. However, L-161,982 completely prevented enhancement of cell viability by PGE_2_ (Fig. 1E), demonstrating that the effects of PGE_2_ are mediated through the Gα_s_-coupled EP4 receptor.

### Cyclooxygenase-2 inhibition attenuates neuroblastoma cell viability

**Fig. 2:**
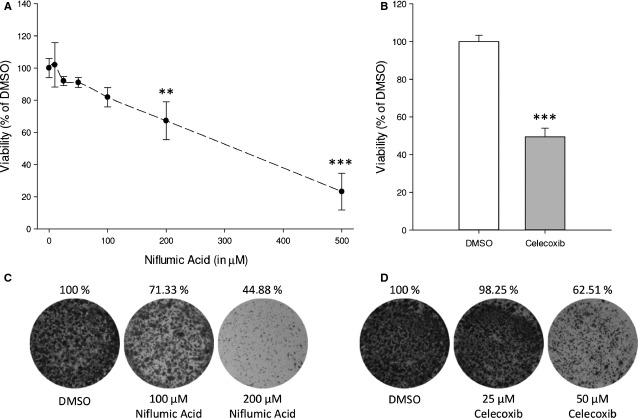
Effect of specific COX-2 inhibition on SK-N-AS cell viability. (**A**) Cell viability after 48-hr incubation with indicated concentrations of niflumic acid (IC_50_ SK-N-AS 355 μM). (**B**) Cell viability after 48-hr incubation with 50 μM celecoxib. (**C** and **D**) Representative pictures of a colony formation assay. Data represent mean ± SE of the mean of 4–10 separate experiments. **P < 0.01, ***P < 0.001 compared to DMSO-treated cells.

Prostaglandin E_2_ is produced by a multistep enzymatic process from arachidonic acid, in which the rate-limiting step is mediated by COX. Expression of COX-2 is increased in many cancers, including neuroblastoma, resulting in high PGE_2_ production [31]. As PGE_2_ enhances cell cycle progression, inhibition of PGE_2_ production by COX-2 inhibition should attenuate cell proliferation. In overnight culture, neuroblastoma cells produce PGE_2_ (150 pg/ml) and COX-2 inhibition with niflumic acid decreases PGE_2_ (6 pg/ml). Importantly, niflumic acid decreases dose-dependently viability of SK-N-AS cells (Fig. 2A). To confirm that the niflumic acid data were the result of COX-2 inhibition, another COX-2 inhibitor was used, celecoxib. Similar to niflumic acid, celecoxib treatment resulted in a marked decrease in cell viability (Fig. 2B). To visualize decreased cell survival over a longer time, cells were cultured for 14 days in a colony formation assay in the presence or absence of either niflumic acid (Fig. 2C) or celecoxib (Fig. 2D). Confluency was markedly decreased in the presence of specific COX-2 inhibitors which was more pronounced when higher concentrations of COX-2 inhibitor were used.

### Cyclooxygenase-2 inhibition blocks cell cycle progression and activates an apoptotic response

**Fig. 3:**
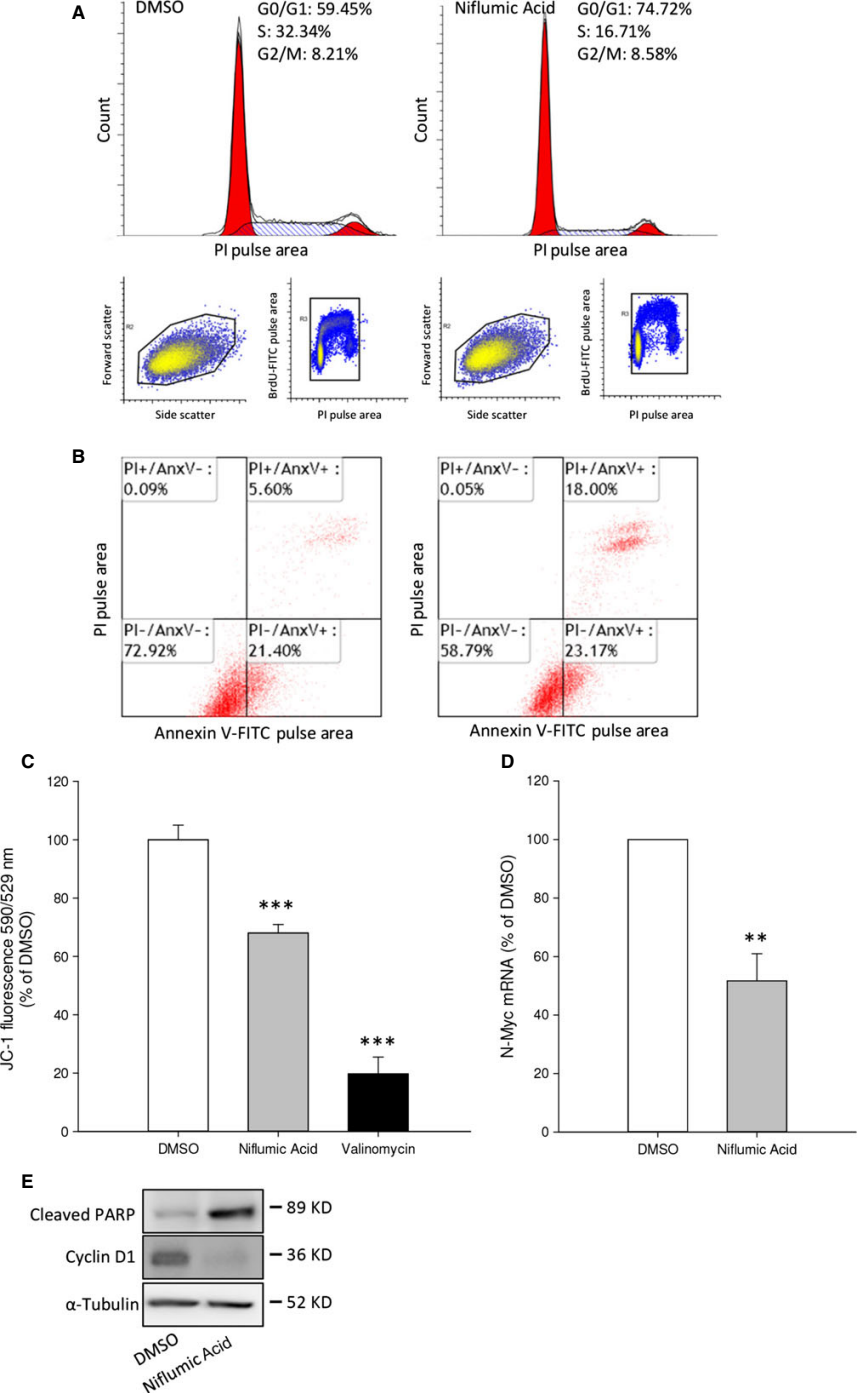
COX-2 inhibition decreases cell cycle progression and induces apoptotic events in SK-N-AS cells. (**A**) Cell cycle analysis after 200 μM niflumic acid. (**B**) Niflumic acid-induced apoptosis was analysed by annexin V labelling. Lower left: viable cells; lower right: early apoptotic cells; upper right: late apoptotic/necrotic cells. (**C**) Mitochondrial membrane polarization was measured with JC-1 in cells incubated with niflumic acid. (**D**) qPCR data showing N-Myc mRNA expression after 16-hr incubation with niflumic acid. (**E**) Representative Western blot images showing niflumic acid-induced PARP cleavage and expression of Cyclin D1 after 24 hrs. Data represent mean ± SE of the mean of 4–10 separate experiments. ***P < 0.001 compared to DMSO-treated cells.

To clarify mechanisms underlying the reduction in cell survival by COX-2 inhibition, we studied cell cycle progression and apoptotic pathway activation. Cell cycle progression was first studied by BrdU incorporation followed by fluorescence-activated cell sorting using cytometry (Fig. 3A). PI was used to measure the overall DNA content of cells. Niflumic acid reduced the amount of BrdU-positive cells and cells with a ploidy between 2n (cells in G0/G1) and 4n (cells in G2/M), indicating a lower fraction of cells in S-phase. In contrast, induction of apoptosis was studied by sorting cells based on membrane expression of annexin V (Fig. 3B). Viable cells are located in the lower left quadrant (double negative), early apoptotic cells are located in the lower right quadrant (annexin V positive, PI negative) and late apoptotic cells are located in the upper right quadrant (double positive). Niflumic acid treatment exerts a higher percentage of cells in late apoptosis, while the percentage of cells in early apoptosis was not affected. To confirm apoptotic pathway activation, we measured aggregation of the JC-1 dye at mitochondrial membranes. A distinctive feature of the early stages apoptosis is the disruption of active mitochondria, which includes changes in the membrane potential. In healthy, polarized membranes, the JC-1 dye spontaneously forms aggregates with red fluorescence (EM 590 nm), while in apoptotic cells, the JC-1 dye remains in its monomeric form (EM 529 nm). Inhibition of COX-2 with niflumic acid results in attenuated mitochondrial membrane polarization (Fig. 3C). Valinomycin served as a positive control.

Amplification and thereby increased expression of N-Myc is the best known marker for neuroblastoma risk profile [1–3]. N-Myc is a positive regulator of cell cycle progression and a negative regulator of programmed cell death. COX-2 inhibition with niflumic acid decreased expression of N-Myc mRNA (Fig. 3D). To further distinguish between inhibition of cell cycle progression and induction of apoptotic signalling, we looked for expression of cyclin D1. Niflumic acid attenuated expression of cyclin D1, which is consistent with the cell cycle analysis shown in Figure 3A. Finally, niflumic acid-induced cleavage of the apoptosis linked Poly ADP ribose polymerase (PARP) (Fig. 3E).

### Rescue of cyclooxygenase-2-dependent cell survival by exogenous prostaglandin E_2_

**Fig. 4:**
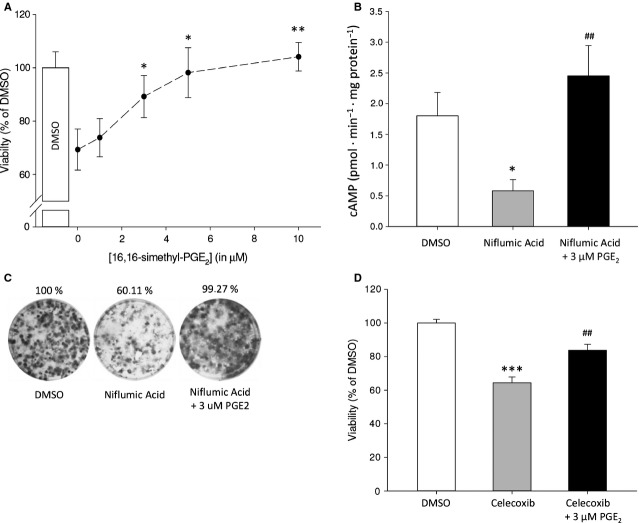
Attenuated cell viability of SK-N-AS cells in response to COX-2 inhibition can be restored by PGE_2_. (**A**) Viability after 48-hr incubation with niflumic acid in the absence or presence of the indicated concentrations of PGE_2_. (**B**) cAMP formation in response to niflumic acid with and without PGE_2_. (**C**) Representative pictures of a colony formation assay. (**D**) Cell viability after 48-hr incubation with 25 μM celecoxib and PGE_2_. Data represent mean ± SE of the mean of four separate experiments. *P < 0.05, **P < 0.01 compared to DMSO-treated cells, ^#^*P* < 0.05, ^##^*P* < 0.01 compared to COX-2 inhibitor-treated cells.

To confirm that the decreased viability of neuroblastoma cells in response to COX-2 inhibition (Fig. 2) is a result of lower PGE_2_ levels, cells were incubated with niflumic acid absence or presence of exogenous PGE_2_. We used a concentration of niflumic acid that results in ±75% viability compared to control-treated cells. Reduced viability resulting from COX-2 inhibition by niflumic acid was completely restored by exogenous PGE_2_ in a dose-dependent manner (Fig. 4A). In addition, niflumic acid treatment resulted in a marked decrease in cAMP production, which was rescued by simultaneous addition of exogenous PGE_2_ (Fig. 4B).

Likewise, exogenous PGE_2_ prevented inhibition of colony formation induced by COX-2 inhibition (Fig. 4C). Similarly, celecoxib attenuated neuroblastoma cell viability, which was restored by exogenous PGE_2_ (Fig. 4D).

### cAMP enhances β-catenin stability and activates β-catenin target gene transcription

**Fig. 5:**
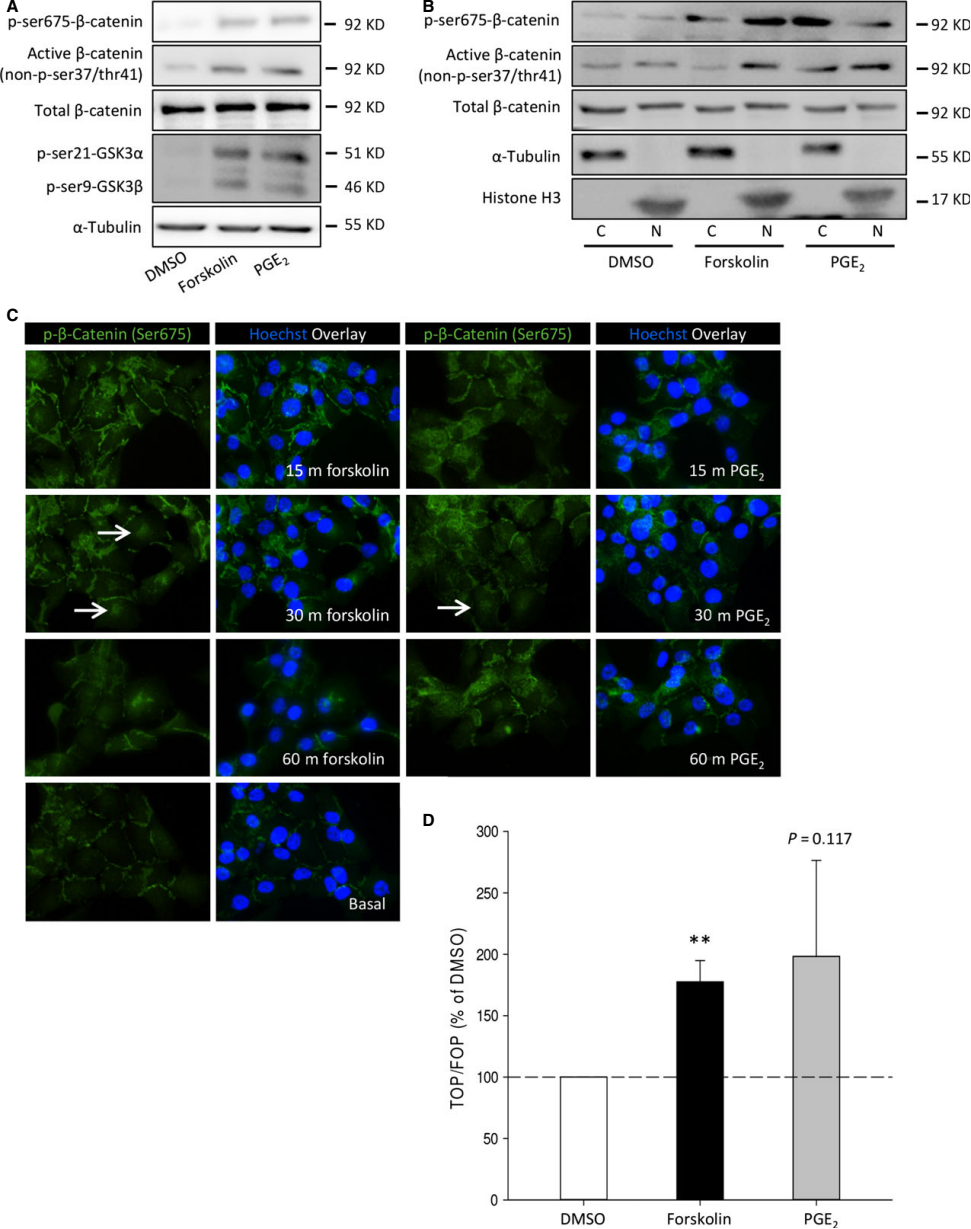
β-Catenin activity is enhanced by PGE_2_ and forskolin in SK-N-AS cells. (**A**) Representative Western blot images of active β-catenin, p-β-catenin (ser675) and p-GSK3β (ser9) in cells incubated with forskolin or PGE_2_ for 30 min. (**B**) Representative Western blot images of cytosolic (C) and nuclear fractions (N) of active β-catenin and p-β-catenin (ser675) in cells incubated with forskolin or PGE_2_ for 30 min. (**C**) Representative immunofluorescence images of p-β-catenin (ser675) in response to forskolin or PGE_2_ for the indicated periods of time. White arrows indicate presence at (peri)nuclear regions. (**D**) TOPFlash assay of cells incubated with forskolin or PGE_2_. Data represent mean ± SE of the mean of four separate experiments. *P < 0.05, ***P < 0.001 compared to DMSO-treated cells.

β-catenin stability and transcriptional activity are known to determine the balance between cell proliferation and differentiation and are involved in the progression of several cancers [32]. Because several recent reports suggest that PGE_2_ can enhance β-catenin stability and transcriptional activity [22,33–36], we aimed to identify the effects of forskolin and PGE_2_ on β-catenin stability and transcriptional activity. In response to cAMP elevation, PKA is activated. PKA can phosphorylate β-catenin and GSK3β (see introduction). β-catenin not phosphorylated by GSK3β is stable and translocates to the nucleus. In this paper, we refer to this stable form of β-catenin as active β-catenin. Neuroblastoma cells stimulated with forskolin or PGE_2_ were analysed for protein levels of phosphorylated β-catenin and GSK3β. Forskolin and PGE_2_ induced phosphorylation of β-catenin (ser675), p-GSK3β (ser9) and concomitant higher levels of active, unphosphorylated, β-catenin (Fig. 5A). It has been reported that phosphorylation of β-catenin by PKA (ser675) enhances β-catenin stability and its nuclear translocation [24,37,38]. β-Catenin localization was determined by analysis of cytosolic (C) and nuclear fractions (N) (Fig. 5B). Phosphorylation of β-catenin (ser675) was increased in both nuclear and cytosolic fractions by cAMP and PGE_2_. Levels of active β-catenin were increased in nuclear fractions by cAMP and PGE_2_. To visualize localization of p-β-catenin (ser675), neuroblastoma cells were stimulated with forskolin or PGE_2_ after which p-β-catenin (ser675) was visualized by immunofluorescence (Fig. 5C). Both forskolin and PGE_2_ transiently increased levels of p-β-catenin (ser675) and induced accumulation of p-β-catenin (ser675) in (peri)nuclear regions (white arrows, Fig. 5C), which was followed by a rapid decline. To assess β-catenin/TCF target gene transcription in response to forskolin or PGE_2_, neuroblastoma cells were transfected with a TCF luciferase reporter gene construct (TOPflash) containing multiple TCF-binding sites or a construct with mutated TCF-binding sites (FOPflash). Either forskolin or PGE_2_ induced TCF reporter gene expression, as measured by luciferase activity in cell lysates (Fig. 5D).

### β-Catenin is involved in neuroblastoma cell survival

**Fig. 6:**
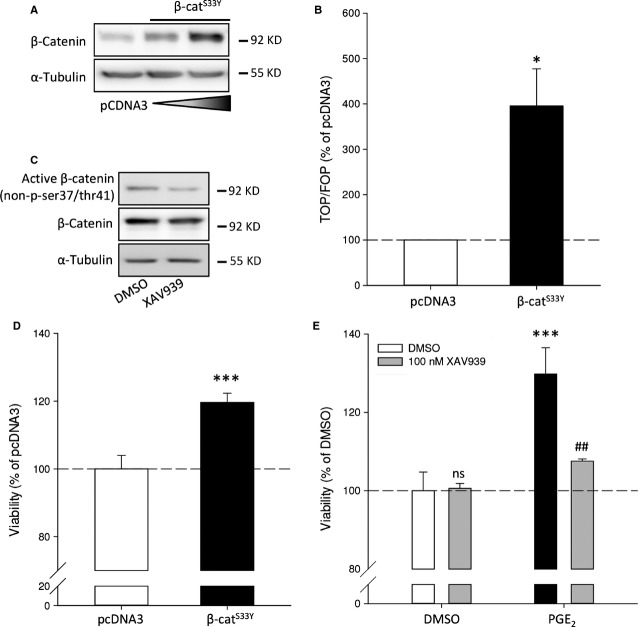
Overexpression of a degradation-resistant mutant of β-catenin (β-catenin^S33Y^) in SK-N-AS cells and inhibition of β-catenin by XAV939. (**A**) Representative Western blot images of β-catenin^S33Y^ expression. (**B**) TOPFlash assay of cells expressing of β-catenin^S33Y^. (**C**) Representative Western blot images of β-catenin expression in cells after 1 μM XAV939 treatment for 24 hrs. (**D**) Cell viability of cells expressing of β-catenin^S33Y^. (**E**) Cell viability of cells incubated with PGE_2_ absence or presence of XAV939. Data represent mean ± SE of the mean of four separate experiments. *P < 0.05, ***P < 0.001 compared to DMSO-treated cells, ^#^*P* < 0.05 compared to PGE_2_-treated cells.

To study if the effects of forskolin and PGE_2_ on cell survival are mediated *via* β-catenin-dependent gene transcription in a non-Wnt ligand-dependent manner, neuroblastoma cells were transfected with the stable active mutant of β-catenin (β-catenin^S33Y^). Transfection with β-catenin^S33Y^ increased protein levels of β-catenin (Fig. 6A). Functionally, β-catenin^S33Y^ increased TCF reporter gene activity (Fig. 6B). In addition, active β-catenin could enhance cellular viability and cell cycle progression. Overexpression of β-catenin^S33Y^ enhanced cell viability compared to cells expressing the empty vector (Fig. 6D).

Conversely, we addressed whether β-catenin is required for PGE_2_-induced neuroblastoma cell viability. To study this, we lowered active (non-phosphorylated) β-catenin by enhancing the stability of axin2, the limiting factor of the APC/GSK3β/CK1 destruction complex that targets β-catenin for proteasomal degradation, with the tankyrase inhibitor XAV939 [39]. Levels of active β-catenin were reduced by XAV939 treatment, while total levels of β-catenin remained unchanged (Fig. 6C). Importantly, while XAV939 prevented PGE_2_-induced cell viability, it did not affect neuroblastoma cell viability at a basal level (Fig. 6E). Thus, our data indicate that the effects of PGE_2_ on neuroblastoma involve active β-catenin.

### SK-N-SH human neuroblastoma cells

**Fig. 7:**
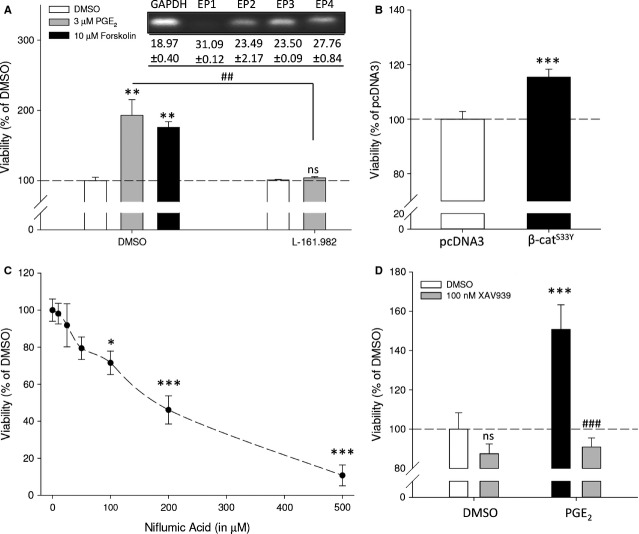
Viability of SK-N-SH human neuroblastoma cells after modulation by PGE_2_ and β-catenin. (**A**) Cell viability after 48 hrs of cells incubated with forskolin or PGE_2_ and L-161,982. (**B**) Cell viability of cells expressing of β-catenin^S33Y^. (**C**) Cell viability after 48-hr incubation with indicated concentrations of niflumic acid. (**D**) Cell viability of cells incubated with PGE_2_ in the absence or presence of XAV939. Data represent mean ± SE of the mean of 4–10 separate experiments. *P < 0.05, **P < 0.01, ***P < 0.001 compared to DMSO-treated cells, ^#^*P* < 0.05 compared to PGE_2_-treated cells.

Cancer cell lines are often characterized by cell line-specific (genetic) abrogations that do not accurately reflect the behaviour of other cancer cell lines originating from the same disease. We therefore repeated key experiments in a different human neuroblastoma cell line without *MYCN* amplification, SK-N-SH. Similar to SK-N-AS, forskolin and PGE_2_ enhanced SK-N-SH neuroblastoma cell viability, while antagonism of the EP4 receptor with L-161,982 prevented PGE_2_-enhanced cell viability which was not affected by antagonism of the EP2 receptor using AH6809 (Fig. 7A and data not shown). In addition, overexpression of β-catenin^S33Y^ enhanced cell viability (Figs 6D and 7B). Specific COX-2 inhibition with niflumic acid or celecoxib (data not shown) attenuated cell viability in a dose-dependent manner (Figs 2A and 7C). SK-N-SH cells were more sensitive towards COX-2 inhibition compared to SK-N-AS. The PGE_2_-induced enhanced cell viability was prevented by attenuating active β-catenin with the tankyrase inhibitor XAV939 (Figs 6E and 7D). Thus, SK-N-SH and SK-N-AS human neuroblastoma cell lines behave similarly in response to manipulation of PGE_2_ or β-catenin signalling properties.

### High-risk neuroblastoma without amplification of *MYCN* exhibit high β-catenin expression

**Fig. 8:**
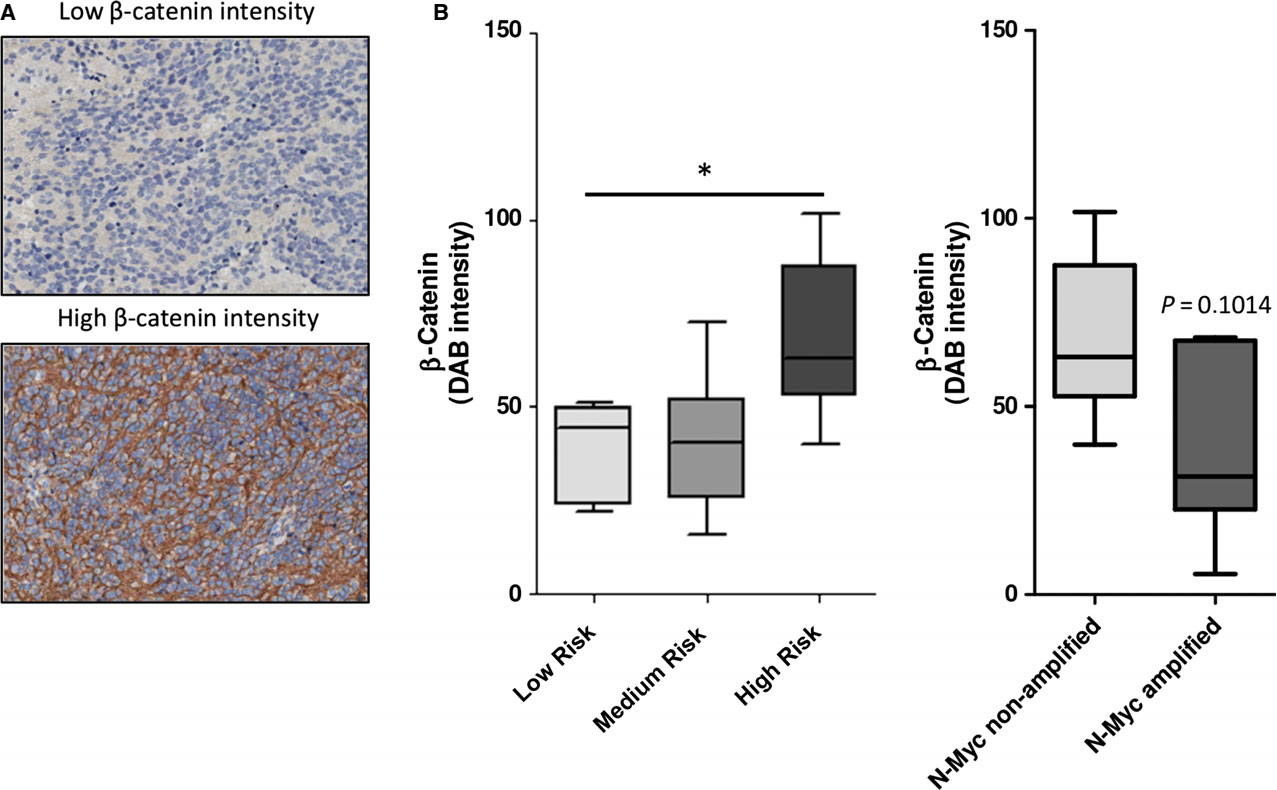
β-Catenin expression is increased in high-risk neuroblastoma tumours without amplification of *MYCN*. (**A**) Characteristics of study population. For this study, we focused on tumours that have no *MYCN* amplification. (**B**) Representative IHC pictures of low and high β-catenin expression. (**C**) Quantification of average β-catenin intensity in tumours without amplification of *MYCN*. Boxes represent Q_1_, median (Q_2_) and Q_3_. Whiskers represent minimum and maximum. *P < 0.05 (Kruskal–Wallis).

Finally, we examined a potential correlation between β-catenin expression in neuroblastoma tumour sections with neuroblastoma risk profile. We investigated tumour specimens that were collected and stored for diagnosis for expression of β-catenin protein by immunohistochemistry. Characteristics of the investigated risk group populations are listed in Table 4. We were only interested in cases without *MYCN* amplification as it has previously been reported that β-catenin expression is increased in neuroblastoma tumours and cell lines without amplification of *MYCN* [16]. Fluorescence *in situ* hybridization was performed to identify amplification status of neuroblastoma specimens. We regarded tumour specimens that scored positive for *MYCN* amplification as a separate group of tumours. β-Catenin expression was determined by scoring the average staining intensity of the tumour. β-Catenin was found predominantly at the periphery of the cell body and within the neuropil, areas of the tumour that are comprised of non-myelinated axons, dendrites and synaptic dense regions. We did not observe β-catenin-positive nuclei. When high-risk tumours with *MYCN* amplification were compared to high-risk tumours without *MYCN* amplification, higher levels of β-catenin were found in high-risk tumours without *MYCN* amplification. Importantly, we observed higher levels of β-catenin in tumour specimens from high-risk tumours compared to expression in low- and medium-risk cases (*P* = 0.042) (Fig. 8C).

**Table 4:** Characteristics of study population

Risk (COG)	*n*	Sex	Age (in months)	Stage (INSS)					Me	St	Pr	Re	De

				1	2a/b	3	4	4S					
Low	7/22	M5/F2	Median: 2 Range: 0–68	2/7	4/7	0/7	0/7	1/7	1/7	2/7	0/7	5/7	1/7
Medium	9/22	M7/F2	Median: 7 Range: 0–12	0/9	0/9	8/9	1/9	0/9	1/9	1/9	0/9	8/9	1/9
High	6/22	M3/F3	Median: 112 Range: 13–267	0/6	0/6	1/6	5/6	0/6	5/6	2/6	2/6	2/6	3/6

COG, Children's Oncology Group; INSS, International Neuroblastoma Staging System; M: male; F: female; Me: metastatic; St: stable; Pr: progressing; Re: remission; De: deceased.

## Discussion

The development of neural crest-derived tissues is under tight control of molecular mechanisms that regulate proliferation, differentiation and programmed cell death. When these core molecular pathways become deregulated, malignancies such as neuroblastoma can develop. The most extensively defined factor contributing to neuroblastoma pathogenesis is amplification of the Myc oncoprotein family member *MYCN*. Amplification of *MYCN* is only observed in about one-fourth of neuroblastomas [1,3]. In this study, we focused on the contribution of PGE_2_ and β-catenin to progression of neuroblastomas without *MYCN* amplification. Our findings in *MYCN* non-amplified neuroblastoma cell lines show that PGE_2_ enhances cell viability through the EP4 receptor, whereas this was attenuated by inhibition of PGE_2_ with specific COX-2 inhibitors. Elevating cAMP using forskolin mimicked the effects of PGE_2_ on cell viability. PGE_2_ and cAMP promoted GSK3β inhibition, phosphorylation of β-catenin at the PKA target residue ser675, β-catenin nuclear translocation and TCF-dependent gene transcription. Furthermore, we show that expression of a degradation-resistant β-catenin mutant enhances neuroblastoma cell viability and that inhibition of β-catenin with XAV939 prevents PGE_2_-induced viability. β-Catenin expression in tumour sections was increased in high-risk neuroblastoma without *MYCN* amplification compared to expression in low- or medium-risk sections. These data suggest that autocrine PGE_2_ enhances neuroblastoma cell viability *via* a mechanism involving cAMP-mediated β-catenin stabilization, and suggest that this pathway is of relevance to high-risk neuroblastoma without *MYCN* amplification.

Increased PGE_2_ levels and concomitant up-regulation of COX-2 are frequently observed in malignant tissues of epithelial origin [5,40]. Studies have revealed that inhibition of COX enzymes have potential in anticancer therapy [40]. Regarding neuroblastoma, studies indicate that COX-2 and PGE_2_ are involved in progression of neuroblastoma [4,5], although it is largely unknown which downstream molecular pathways are involved. PGE_2_ functions by binding to its membrane bound E-type prostanoid receptors which are all expressed in neuroblastoma cells. Of the four receptor subtypes, EP2 and EP4 are Gα_s_-coupled and stimulate adenylyl cyclase activation and thus intracellular cAMP levels [41]. Indeed, we found that PGE_2_ incubation elevated cellular cAMP levels in neuroblastoma. Conversely, inhibiting endogenous PGE_2_ synthesis with COX-2 inhibitors decreased cAMP levels. The rise in cAMP levels, either through receptor-mediated adenylyl cyclase activation by PGE_2_ or *via* direct activation of adenylyl cyclase by forskolin, increased cell viability of neuroblastoma cells. Further, we demonstrate that enhancement of cell viability by PGE_2_ is mediated through the EP4 receptor as antagonism of the EP4 receptor using L-161,982 completely abolished the enhancement of cell viability by PGE_2_, whereas antagonism of the EP2 receptor using AH6809 did not affect this. In earlier studies, Rasmuson *et al*. demonstrated that exogenous PGE_2_ enhanced viability of *MYCN*-amplified SK-N-BE(2) neuroblastoma cells, suggesting that PGE_2_ is involved in neuroblastoma cell viability independent of amplification of *MYCN* [4].

Inhibition of COX enzymes has been shown to inhibit neuroblastoma cell survival *in vitro* [7,42] and to inhibit tumour growth *in vivo* [5]. Indeed, COX-2 inhibitors attenuated cell viability in neuroblastoma cells. In other studies, inhibition of COX-2 by celecoxib or diclofenac changed mitochondrial membrane potential and subsequent activation of caspase-9- and caspase-3-dependent apoptosis [7,43], while no activation of caspase-8 or BID cleavage were observed, suggesting involvement of the intrinsic apoptotic pathway. In this study, we observed altered mitochondrial membrane potential after treatment with COX-2 inhibitors. In addition, we found that COX-2 inhibition induces apoptosis in neuroblastoma cells. Furthermore, we observed attenuated cell cycle progression after COX-2 inhibition resulting from a cell cycle block between G1 and S-phase. This cell cycle block was further confirmed by decreased expression of cell cycle regulatory protein cyclin D1. Although it seems evident that the effects of COX-2 inhibition are mediated by decreased PGE_2_ production, there are several reports that indicate that accumulation of arachidonic acid, the precursor for PGE_2_ synthesis, is also involved. For instance, combined treatment with arachidonic acid and COX inhibitors results in a synergistic effect on cell survival [44]. However in this study, we show that COX-2 inhibition with niflumic acid or celecoxib complemented with exogenous PGE_2_, rescues neuroblastoma cells from decreased viability as a result of COX-2 inhibition. This is also reflected on intracellular cAMP levels, which are decreased by niflumic acid, but restored by simultaneous addition of exogenous PGE_2_.

Of particular interest are emerging research lines that focus on molecular interactions between PGE_2_ and β-catenin. In 2002, Fujino *et al*. showed that PGE_2_ stimulates TCF/Lef promoter activity through EP2 and EP4 receptor signalling [33]. Downstream of EP2 and EP4, an increase in cAMP levels activates PKA. Phosphorylation of GSK3β at ser9 by PKA inhibits its kinase activity and thereby enhances β-catenin stability, nuclear translocation and TCF/Lef-dependent gene transcription [23,32,33]. It was, however, unknown if this molecular interaction occurs in neuroblastoma. Stimulation of neuroblastoma cells with forskolin or PGE_2_ increased p-GSK3β (ser9) and elevated levels of unphosphorylated β-catenin at the GSK3β residues. In addition, we found increased nuclear localization of active β-catenin. These findings are in agreement with studies in different malignant cell models. In non-small cell lung cancer and colorectal cancer, PGE_2_ enhances β-catenin nuclear localization and inhibits GSK3β by phosphorylation which is dependent on cAMP and kinase activity of PKA [34,36]. Apart from inhibiting GSK3β, PKA has been shown to directly activate β-catenin by phosphorylation at residue ser675 [24,37,38]. Indeed, we observed increased p-β-catenin (ser675) in response to PGE_2_ or forskolin. Subsequently, we observed a transient increase in p-β-catenin (ser675) at the (peri)nuclear regions. Importantly, we also found increased TCF-dependent gene transcription, which confirms earlier studies in HEK cells in which PKA-dependent p-β-catenin (ser675) resulted in increased TCF luciferase activity [24,37]. More recently, increased TCF-dependent gene transcription downstream of PGE_2_ and subsequent PKA activity was found in *in vitro* and *in vivo* models for colorectal cancer [23,34]. Conversely, inhibition of PGE_2_ synthesis using the COX-2 inhibitor celecoxib has been found to attenuate β-catenin stability, nuclear translocation and TCF luciferase activity in colorectal cancer [34,35], non-small cell lung cancer [36], and osteosarcoma [45], with concomitant lower expression of cyclin D1 and cell survival.

The contribution of β-catenin and TCF-dependent gene transcription in colorectal carcinoma has been well-established. In neuroblastoma, it has been shown that β-catenin expression is increased in *MYCN* non-amplified neuroblastoma cell lines [16]. The result of β-catenin transcriptional activity is expression of mitogenic and survival genes. In this study, we demonstrate increased TCF-dependent gene transcription and enhanced cellular viability in neuroblastoma cells transfected with a β-catenin mutant that is untargetable for proteasomal degradation (β-catenin^S33Y^). Importantly, the enhanced cell viability resulting from PGE_2_ was completely prevented by the tankyrase inhibitor XAV939. XAV939 prevents tankyrase-mediated degradation of axin2, thereby stabilizing axin2 which is the concentration-limiting component of the destruction complex that sequesters β-catenin and GSK3β. Thereby, XAV939 functions as an inhibitor of β-catenin activity [39]. Thus, β-catenin is, at least to some extent, responsible for PGE_2_-induced enhanced cellular viability. Recently, XAV939 has been shown to decrease neuroblastoma cell survival by inhibiting β-catenin transcriptional activity and decreasing expression of cyclin D1 in both *MYCN*-amplified and non-amplified cell lines [46]. Moreover, knockdown of β-catenin using RNA interference has been shown to inhibit cell cycle progression and induce apoptosis in neuroblastoma cells [47]. This, together with our data indicates that β-catenin plays a crucial role in neuroblastoma cell survival.

Interestingly, investigation of β-catenin expression in neuroblastoma tumour sections without *MYCN* amplification revealed particularly high expression in high-risk tumours. This further strengthens our hypothesis that β-catenin plays an important role in neuroblastoma, not only *in vitro* cell models but also in a clinically more relevant context. Other recent studies have also found increased expression of β-catenin in neuroblastoma, which was involved in maintaining a neuroblast-like phenotype of neuroblastoma cells and conferring resistance to chemotherapeutic agents [48,49]. However, these studies did not distinguish between *MYCN*-amplified and non-amplified tumours. We are aware that based on our study, we cannot draw strong conclusions on the functional role of increased β-catenin in high-risk neuroblastoma tumours as expression of β-catenin was found predominantly at the cell periphery and the neuropil, where it exerts a different function than in the nucleus. However, during epithelial–mesenchymal transition, which is an early step in the metastatic cascade in cancer [50], β-catenin is liberated from the junctional complexes where it is sequestered. In such an event in the presence of a β-catenin stabilizing stimulus such as PGE_2_, β-catenin translocates to the nucleus and activates TCF/Lef-dependent transcriptional programmes, thereby enhancing survival and metastatic potential [51].

The promising therapeutic potential of COX-2 inhibitors has resulted in multiple compounds that have reached the clinical market. These compounds were, however, rejected as a consequence of concerns regarding drug safety [40]. Recent insight in the systemic effects of COX-2 inhibition and its metabolites, questions the notion that the safety issues are the result of COX-2 inhibition and not COX-1 [52,53]. We believe that with better insights in the molecular events that occur downstream of COX inhibition, more specific chemopreventive compounds might be developed that exploit the therapeutic potential of specific COX-2 inhibition, but circumvent the safety issues.

Taken together, our results demonstrate that PGE_2_ is an important factor being involved in the regulation of neuroblastoma cell viability. cAMP activates PKA and thereby stabilizes β-catenin either directly or indirectly by inhibiting GSK3β, resulting in increased β-catenin activity and neuroblastoma cell viability. The increased expression of β-catenin in high-risk neuroblastoma, without *MYCN* amplification, suggests that β-catenin could play a major role in neuroblastoma. Thus, strategies that target either PGE_2_ production by the tumour (COX-2 inhibitors) or target the interaction between PGE_2_ and β-catenin warrant further investigation.
